# Regulatory T cells in Crohn's disease following anti‐TNF‐α therapy

**DOI:** 10.1002/jgh3.12259

**Published:** 2019-09-11

**Authors:** Toshimi Chiba, Mikiya Endo, Shoko Miura, Yuko Hayashi, Yoshiko Asakura, Kotaro Oyama, Takayuki Matsumoto

**Affiliations:** ^1^ Division of Internal Medicine, Department of Oral Medicine Iwate Medical University Morioka Japan; ^2^ Department of Pediatrics Iwate Medical University Morioka Japan; ^3^ Division of Gastroenterology, Department of Internal Medicine Iwate Medical University Morioka Japan

**Keywords:** anti‐TNF‐α therapy, CD127^−^, Crohn's disease, FoxP3^+^, Tregs

## Abstract

**Background and Aim:**

Anti‐tumor necrosis factor alpha (TNF‐α) therapy is an effective therapy for Crohn's disease (CD). We investigated FoxP3^+^ and CD127^−^ regulatory T cells (Tregs) before and after administration of anti‐TNF‐α therapy in CD.

**Methods:**

Eight patients with active CD who had received anti‐TNF‐α antibodies were enrolled. Treatment responses were followed by physical examination and Crohn's disease activity index (CDAI) scoring before and 2 weeks after the initial administration of anti‐TNF‐α antibodies. Peripheral blood samples were collected before and 2 weeks after treatment. White blood cell count and serum levels of C‐reactive protein (CRP) and albumin were measured. FoxP3^+^ expression and CD127^−^ Tregs were measured by fluorescence activated cell sorting (FACS) analysis of whole blood samples.

**Results:**

Median values of CDAI decreased significantly after treatment. The proportion of FoxP3^+^ Tregs increased significantly after treatment. There was a significant negative correlation between ΔCD127^−^ Tregs and Δlymphocyte.

**Conclusions:**

Anti‐TNF‐α therapy would enhance Tregs, which may account for the mechanism underlying the positive effect of the anti‐TNF‐α treatment in CD patients.

## Graphical abstract

We investigated regulatory T cells before and after administration of anti‐TNF‐a therapy in Crohn's disease. The proportion of FoxP3+ Tregs increased significantly after treatment. There was a significant negative correlation between ΔCD127^−^ Tregs and Δlymphocyte.

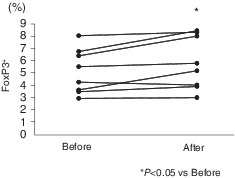

## Introduction

Anti‐tumor necrosis factor alpha (TNF‐α) therapy has been reported to be effective for induction and maintenance therapy for Crohn's disease (CD). Regulatory T cells (Tregs) are characterized by the expression of CD4, CD25, and the transcription factor forkhead box P3 (FoxP3). Treg function is necessary to protect against autoimmunity and intestinal inflammation and is dependent on suppressive cytokines, transforming growth factor‐β (TGF‐β), and interleukin (IL)‐10.^1^, ^2^ IL‐7 receptor (CD127) is a biomarker of Tregs in human peripheral blood as the expression of FoxP3 and CD127 is inversely correlated within CD4^+^CD25^+^ T cells.^3^, ^4^

FoxP3+ Tregs spread and collect on the inflamed mucosa of the inflammatory bowel disease (IBD) patients.^5^, ^6^ T helper cells produce IL‐17 (Th17 cells) for mucosal homoeostasis and are implicated in the pathogenesis of CD.^7^, ^8^ Th17 cells would protect the host from infection at mucosal surfaces, whereas Tregs control immune responses and inflammation caused by the microflora.^9^, ^10^

A reduced ratio of Tregs in the peripheral blood of IBD has been reported, whereas FoxP3 mRNA levels in the mucosa are elevated in IBD.^11^ Tregs are also increased in the lamina propria (LP) and decreased in blood in CD patients.^12^, ^13^, ^14^, ^15^ Tregs have also been significantly increased after infliximab (IFX) treatment.^16^, ^17^, ^18^

In the present study, we examined FoxP3^+^ and CD127^−^ Tregs in CD patients before and after administration of anti‐TNF‐α therapy to determine if FoxP3^+^ and CD127^−^ Tregs correlate with the perpetuation of CD and to examine the mechanism of action of anti‐TNF‐α therapy.

## Methods

### *Patients*

Eight patients with active CD (five males and three females; mean age ± SD, 33.6 ± 15.1 years) were included in the study. One patient had ileal disease, six patients had ileocolonic disease, and 1 patient exhibited colonic disease. Four patients had anal disease. Disease duration ranged from 1 to 12 years, with a mean of 4.0 years.

### *Anti‐TNF‐α treatment*

Either IFX was administered intravenously at a dose of 5 mg/kg or adalimumab (ADA) was administered subcutaneously at a starting dose of 160 mg. Treatment responses were followed by physical examination and Crohn's disease activity index (CDAI) scoring before and 2 weeks after the initial administration of anti‐TNF‐α. Peripheral blood samples were collected before and 2 weeks after treatment. White blood cell (WBC) count, blood lymphocyte (Lymph) and serum levels of C‐reactive protein (CRP), total protein (TP), and albumin (Alb) were measured.

### *Quantitative measurement of FoxP3^+^ and CD127^−^ Tregs*

Blood mononuclear cells were isolated within 6 h of blood sampling and then frozen at −80°C. For analysis, frozen blood mononuclear cells were thawed in a 37°C water bath. Then, blood mononuclear cells were stained in media with the following antihuman monoclonal antibodies and conjugates for 20 min at room temperature: CD4‐FITC, CD25‐PE‐Cy5, and CD127‐PE. Intracellular staining was performed with FoxP3‐PE monoclonal antibody. All stained samples were examined on a FACSCalibur flow cytometer, and data were analyzed with CellQuestPro software (BD Biosciences, San Jose, CA, USA) to determine CD4^+^CD25^+^FoxP3^+^ (FoxP3^+^ Treg) or CD4^+^CD25^+^CD127^−^ (CD127^−^ Treg).

### *Statistical analysis*

FoxP3^+^ and CD127^−^ Tregs, CDAI, and CRP data are expressed as median values. The Wilcoxon signed‐ranks test was used to compare the median values of proportions of Tregs between pre‐ and postadministration of anti‐TNF‐α time points. The correlation between the proportions of FoxP3^+^ and CD127^−^ Tregs was evaluated using Pearson's correlation test. A *P* value <0.05 was considered to be statistically significant.

## Results

### *Efficacy of anti‐TNF‐α therapy*

**Figure 1:**
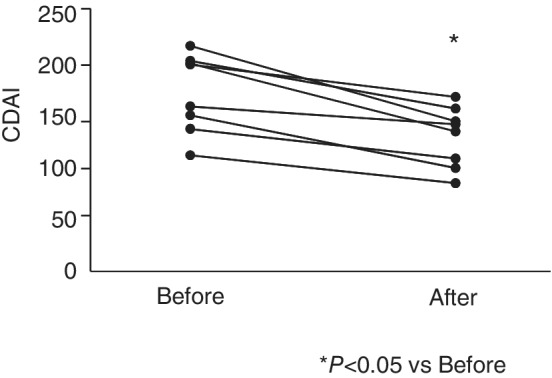
Crohn's disease activity index (CDAI) scores before and after anti‐TNF‐α treatment. Median values of CDAI after treatment were significantly decreased compared to before treatment (*P* < 0.05).

Median values of CDAI after treatment were significantly decreased compared to values before treatment (from 200.5 to 142.2, *P* < 0.05) (Fig. 1). There were no statistically significant differences in serum CRP values, serum protein, serum albumin, white blood count, and blood lymphocyte between pre‐ and postadministration of anti‐TNF‐α antibody samples.

### *Expression of FoxP3^+^ and CD127^−^ Tregs before and after anti‐TNF‐α therapy*

**Figure 2:**
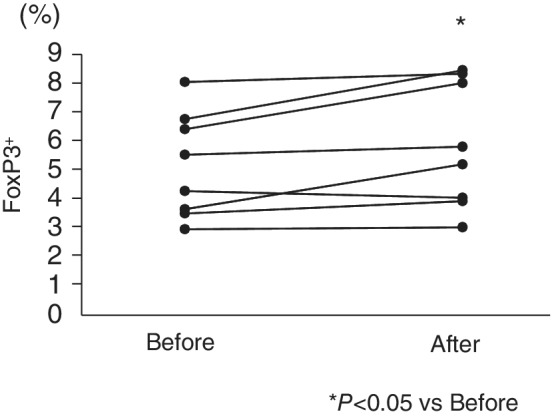
Expression of FoxP3^+^ Tregs before and after anti‐TNF‐α treatment. The proportions of FoxP3^+^ Tregs were significantly increased after treatment.

The proportion of FoxP3^+^ Tregs significantly increased after treatment (Fig. 2), whereas the proportion of CD127^−^ Tregs after treatment was not significantly different. There was a significant negative correlation between delta (Δ) CD127^−^ Tregs (defined as the value after treatment—value before treatment) and ΔLymph (*r* = −0.85, *P* = 0.03). There was no significant correlation between foxp3 ^+^ Tregs and CDAI, CRP, WBC, Lymph, TP, and Alb (Table 1), nor was there any significant correlation between cd127 ^−^ Tregs and CDAI, CRP, WBC, TP, and Alb (Table 2).

**Table 1:** Correlation between FOXP3^+^ Tregs and CDAI, CRP, WBC, Lymph, TP and Alb

FoxP3	*r*	*P* value
CDAI	0.58	0.17
CRP	0.58	0.22
WBC	−0.38	0.46
Lymph	−0.71	0.11
TP	−0.55	0.26
Alb	−0.72	0.11

CDAI, Crohn's disease activity index; CRP, C‐reactive protein; TP, total protein; WBC, White blood cell.

**Table 2:** Correlation between CD127^−^ Tregs and CDAI, CRP, WBC, Lymph, TP and Alb

CD127	*r*	*P* value
CDAI	0.66	0.11
CRP	0.68	0.14
WBC	0.11	0.83
Lymph	−0.85	0.03
TP	−0.71	0.11
Alb	−0.79	0.06

CDAI, Crohn's disease activity index; CRP, C‐reactive protein; TP, total protein; WBC, white blood cell.

The study proposal was reviewed and approved by the Human Ethics Review Committee of Iwate Medical University. Written informed consent was obtained from each patient prior to enrollment.

## Discussion

In this study, we observed that the ratio of FoxP3^+^ Tregs was significantly increased after anti‐TNF‐α treatment. A significant increase in Tregs was observed in CD after anti‐TNF‐α therapy.^18^ FoxP3^+^ cells in blood were increased after anti‐TNF‐α treatment.^17^ FoxP3^+^ Treg frequency was lower in patients with active IBD, a significant increase in the circulating frequency of Tregs after anti‐TNF‐α infusion therapy, which paralleled a reduction of IBD.^17^

Furthermore, anti‐TNF‐α therapy yielded a significant and sustained relative increase in peripheral blood Tregs, a change in CRP levels, and durable clinical response.^19^ In the intestinal mucosa of CD patients, an upward trend in the tissue levels of Tregs after biological therapy has been observed,^20^ similar to the increase in the frequency of Tregs and FoxP3 expression in CD patients treated with IFX,^21^ whereas IFX therapy suppressed mucosal mRNA and protein expression of FoxP3 in ulcerative colitis (UC) and CD;^19^, ^22^ in patients with active IBD, anti‐TNF‐α treatment rapidly enhances the frequency of functional FoxP3^+^ Tregs in blood.^17^ The increased apoptosis of local FoxP3^+^ Tregs in IBD inflamed mucosa were exhibited with a reduced frequency and increased apoptosis of peripheral blood Tregs.^23^ After administration of anti‐TNF‐α antibodies, a decrease in the apoptosis of Tregs was shown to correlate closely with an increase in peripheral Treg cell numbers. The correlations between age of patients and duration of disease and Treg frequencies were reported, and the CDAI negatively correlated with Treg frequencies.^20^ We demonstrated that there was a significant negative correlation between ΔCD127^−^ Tregs and ΔLymph. Our findings indicate that the peripheral Treg number was increased in correlation to a reduction of serum number of lymphocyte after anti‐TNF‐α treatment in CD.

The reduced number of peripheral blood Tregs and the increased numbers of peripheral Th17 cells were observed in IBD patients,^11^, ^24^ whereas FoxP3 mRNA expression levels in the mucosa are elevated, and elevated IL‐17A, IL‐1β, and IL‐6 mRNA levels are also elevated in IBD.^11^ Tregs suppress colonic inflammation by downregulating Th17 through TGF‐β.^15^, ^25^

The gut microbiome would be important for the development of IL‐10 and induced FoxP3^+^ Tregs peripherally.^26^, ^27^, ^28^ Clostridium bacteria might be potent inducers of FoxP3^+^ Tregs in the colonic mucosa.^29^ CD4^+^CD8^+^ αα (DP8α) colonic Tregs produced IL‐10 under the influence of *Faecalibacterium prausnitzii*, and DP8α lymphocytes in the LP and peripheral blood were lower in IBD, suggesting that DP8α Tregs may control or prevent inflammation of IBD.^30^

CD4^+^CD25^+^CD127^lo^CD45RA^+^ Tregs might be a candidate for a Treg therapy for CD in future clinical trials.^31^ Tregs have been proposed as a potential therapy for IBD, but the remission and side effects of novel IBD therapies such as long‐term maintain therapies are still unknown.^32^ The first trial of the effectiveness of Tregs for CD recently reported 40% of patients improved after 8 weeks of clinical administration, including a decrease of CRP and fecal calprotectin.^33^ Then, Tregs might be promising candidates for the treatment of CD.

Thus, further studies are required to verify the association between Tregs and anti‐TNF‐α therapy in CD, and we suggest a prospective study.

In conclusion, we have shown that anti‐TNF‐α therapy increased blood FoxP3^+^ Tregs in patients with CD. In addition, there was a significant negative correlation between CD127^−^ Tregs and Lymph. Based on these observations, we conclude that anti‐TNF‐α therapy would enhance Tregs, which may account for the mechanism underlying the efficacy of this immunosuppressive therapy in CD.
